# Preventive administration of shengmaiyin: a novel approach to counteract heatstroke-induced coagulopathy in rats

**DOI:** 10.3389/fphar.2025.1530371

**Published:** 2025-03-14

**Authors:** Longping He, Zhuqing Luo, Lichun Zhang, Xingping Deng, Lincui Zhong, Qingwei Lin, Qingbo Zeng, Ye Zhou, Jingchun Song

**Affiliations:** ^1^ Intensive Care Unit, The 908th Hospital of Chinese PLA Logistic Support Force, Nanchang, China; ^2^ Intensive Care Unit, Changcheng Hospital Affiliated to Nanchang University, Nanchang, China; ^3^ Intensive Care Unit, Nanchang Hongdu Traditional Chinese Medicine Hospital, Nanchang, China

**Keywords:** shengmaiyin, exertional heatstroke, coagulopathy, proteomics, Xpnpep2

## Abstract

**Background:**

Coagulation disorders play a pivotal role in the elevated mortality rates associated with exertional heatstroke (EHS).

**Purpose:**

To investigate the impact of Shengmai Yin Oral Liquid (SMY) on heatstroke-induced coagulopathy (HIC) in rats with EHS and to elucidate the underlying mechanisms.

**Methods:**

A cohort of eighteen male SPF-grade SD rats, each implanted with a telemetric temperature capsule, were randomly allocated to three groups: a normal control (NC) group, an EHS group, and an SMY group (n = 6 per group). The SMY group received SMY orally at a dosage of 20g/(Kg·day) for a period of five consecutive days. Both the EHS and SMY groups were subjected to exercise in a climate-controlled chamber maintained at 40°C with 70% relative humidity until signs of exhaustion and a core body temperature of 42°C were reached, with the duration and distance of their exercise being meticulously documented. Histopathological assessments were performed on the liver, kidney, lung, duodenum, and heart of the rats. Blood samples were collected to measure prothrombin time (PT), activated partial thromboplastin time (APTT), platelet count, and levels of lactic acid (Lac), thrombomodulin (TM), thrombospondin-1 (TSP-1), von Willebrand factor (vWF), and plasminogen activator inhibitor-1 (PAI-1). Plasma samples were subjected to data-independent acquisition (DIA)-based quantitative proteomics analysis, and differentially expressed proteins identified were further authenticated using parallel reaction monitoring (PRM) and Enzyme-Linked Immunosorbent Assay (ELISA).

**Results:**

The SMY group exhibited a significantly extended running distance and time before reaching a core temperature of 42°C compared to the EHS group. Histopathological examination revealed thrombosis in the liver, kidney, lung, duodenum, and heart of rats in the EHS group, whereas no significant thrombosis was observed in the SMY group. The EHS group showed significantly prolonged PT and APTT, increased Lac, decreased platelet count, and elevated plasma levels of TM, vWF, TSP-1, and PAI-1 compared to the NC group (*P* < 0.05). In contrast, the SMY group demonstrated a significant reduction in APTT, an increase in platelet count, and decreased plasma levels of TM, vWF, PAI-1, and TSP-1 compared to the EHS group (*P* < 0.05). Among the 1,189 proteins identified, 56 differentially expressed proteins (DEPs) were associated with SMY’s protective effects against HIC, primarily involved in the upregulation of the relaxin signaling pathway, protein digestion and absorption, platelet activation, and ECM-receptor interaction signaling pathways, as well as the downregulation of the spliceosome and ribosome signaling pathways. PRM quantitative analysis indicated that SMY may upregulate the expression of Nucleobindin-1 (Nucb1), Procollagen C-endopeptidase enhancer 1 (Pcolce), and lectin galactoside-binding soluble 1 (Lgals1), and downregulate the expression of Xpnpep2. Subsequent ELISA validation confirmed a significant increase in plasma Xpnpep2 levels in EHS rats, an effect that was substantially reduced by pre-treatment with SMY.

**Conclusion:**

SMY demonstrates the capacity to mitigate HIC by lessening the impact of vascular endothelial damage and moderating the consumption of coagulation factors and platelets. This salutary influence is correlated with the downregulation of XPNPEP2 expression.

## 1 Background

EHS is a severe medical condition that arises from a critical imbalance between heat generation and heat dissipation during intense physical exertion, culminating in hyperthermia, unconsciousness, and a cascade of multi-organ failure (Liu et al., 2020). Epidemiological studies have indicated that coagulopathy affects over 40% of individuals suffering from EHS, with a staggering mortality rate exceeding 50% (Wang et al., 2021). During an episode of heatstroke, there is a marked elevation in core body temperature, which can precipitate endothelial cell damage within blood vessels, trigger the release of substantial quantities of tissue factor, provoke a massive thrombin activation, and deplete coagulation substrates, culminating in coagulation disorders and potentially progressing to disseminated intravascular coagulation (DIC) (Knapik and Epstein, 2019; Huisse et al., 2008). Consequently, mitigating the coagulopathy induced by EHS is a pivotal strategy to enhance the survival and recovery prospects of patients afflicted by this condition (Hifumi et al., 2018; Zhong et al., 2021).

Shengmaiyin (SMY), a traditional Chinese medicine formula, is a blend of Panax ginseng (Radix Ginseni), Ophiopogon japonicus, and Schisandra chinensis. SMY can be made traditionally with herbs or taken as ready-to-use modern medicines like liquid, capsules, or injections. It is renowned for its properties in invigorating vital energy and nourishing body fluids, and is frequently prescribed for conditions such as heart failure, myocarditis, and arrhythmias (Zhou et al., 2014; Wang et al., 2022). Despite its established therapeutic uses, there is a paucity of research exploring SMY’s potential in preventing coagulation dysfunction associated with EHS. Data-independent acquisition (DIA) mass spectrometry is an innovative proteomics technique that leverages mass spectrometry to quantify and identify a vast array of proteins within complex biological samples. This method offers superior sensitivity, an extended dynamic range, and heightened precision compared to conventional mass spectrometry approaches, providing a more nuanced understanding of protein functions and regulatory mechanisms within biological systems (Meng et al., 2021). The present study is designed to harness the power of DIA quantitative proteomics to elucidate the preventative effects of SMY on EHS-related coagulation dysfunction and to uncover the molecular mechanisms at play.

## 2 Materials and methods

### 2.1 Experimental animals

This study was conducted in strict accordance with the guidelines of the Institutional Animal Care and Use Committee at the No. 908th Hospital of the Chinese People’s Liberation Army (IACUC Approval No. 908yyLL2023075), and it followed the “replacement, reduction and refinement” principle (Flecknell, 2002). A cohort of eighteen healthy adult male Sprague-Dawley rats, aged 7 weeks and weighing between 180 and 200 g, were sourced from Changsha Tianqin Biotechnology Co., Ltd. (License No. SCXK (Xiang) 2019-0014). The animals were housed under standardized conditions, with a controlled temperature range of 22°C–25°C and relative humidity levels between 40% and 60%. They were provided with *ad libitum* access to a nutritionally balanced diet and water, and maintained on a 12-h light-dark cycle to simulate a natural day-night rhythm (lights on from 7 a.m. to 7 p.m.). Prior to the commencement of the experimental procedures, the rats were allowed a 1-week acclimatization period to ensure their wellbeing and minimize the stress associated with the new environment. The rats received humane care according to the Guide for the Care and Use of Laboratory Animals (National Institutes of Health publication 86-23, 1985 revision).

### 2.2 Drugs and reagents

Shengmai Yin Oral Liquid (Drug approval number: Z11020372; Batch number: 23260010) was provided by Tong Ren Tang Pharmaceutical Factory Co., Ltd. Commercially available ELISA kits for the quantification of rat TM, TSP-1, vWF, and PAI-1 were procured from Enzyme-Linked Biotechnology Co., Ltd. (Shanghai, China). ELISA kits for the quantification of rat Lgals1, Nucb1, Pcolce, and Xpnpep2 were obtained from Jiangsu Jingmei Biological Technology Co., Ltd. (Yancheng, China). The BCA (Bicinchoninic Acid) assay kit was obtained from Biyuntian Biotechnology Co., Ltd. (Shanghai, China). Additionally, 0.1% formic acid and 2% acetonitrile were acquired from Gaojing Fine Chemical Co., Ltd. (Hangzhou, China).

### 2.3 Experimental grouping

Following the implantation of telemetry temperature capsules, the eighteen SPF male Sprague-Dawley rats were allowed a 1-week recovery period post-surgery. Subsequently, they were randomly assigned to three experimental groups: NC group (n = 6), EHS group (n = 6), and SMY group (n = 6). The SMY group received a pre-treatment with Shengmai Yin Oral Liquid at a dosage of 20 g/(Kg·day) via oral gavage for a period of five consecutive days, as referenced in previous studies (Wang et al., 2005). The NC and EHS groups were administered an equivalent volume of physiological saline solution by oral gavage over the same duration.

### 2.4 Telemetry temperature capsule implantation

One week prior to the induction of the experimental model (Leon et al., 2004), all rats were prepared for the implantation surgery (Sv-223, implantable telemetry temperature capsule, Shenzhen, China). They were fasted for a period of 24 h, with access to a small amount of water, and were encouraged to defecate. Thirty minutes prior to the procedure, the rats were weighed. Anesthesia was induced using an intraperitoneal injection of pentobarbital sodium at a dosage of 45 mg/kg. The rats were then positioned on the surgical platform, and the surgical site was disinfected. A 1-2 cm incision was made along the anterior midline of the abdominal cavity. The sterilized telemetry temperature capsule was implanted into the abdominal cavity of each rat. The incision was closed in layers, and the surgical site was disinfected post-operatively. Daily monitoring of the rats’ wound healing was conducted following the surgery.

### 2.5 Establishment of EHS model in rats

Prior to model establishment, rats were subjected to a 24-h fasting period. The artificial climate chamber (KW-PT-WS, Nanjing, China) was adjusted to an ambient temperature of 40°C with a relative humidity of 70%. Rats were positioned on a level animal treadmill (KW-PT, Nanjing, China), initiating exercise at a pace of 5 m/min, which was incrementally increased by 1 m/min every 2 min until it stabilized at 15 m/min. Electrodes were strategically placed at the termination of each track, administering an electrical stimulus of 1 mA to compel continuous running. Rats were categorized as exhausted if they ceased running for a duration of 5 s despite the electrical stimulus. Core body temperature was monitored continuously throughout the exercise. The EHS model was deemed successfully established upon the manifestation of exhaustion and an attainment of a core temperature threshold of 42°C (Lin et al., 2021; Fang et al., 2024).

### 2.6 Assessment of coagulation parameters

Rats were anesthetized by isoflurane inhalation for blood collection from abdominal aorta. Blood samples (nine volumes) were collected into tubes containing one volume of sodium citrate (Kangshi, Zhejiang, China), immediately centrifuged at 3000 g for 10 min at room temperature, then assayed using routine blood coagulation tests within 2 h after sampling. PT and APTT were measured using an automatic coagulation analyzer (TOP700, Wolfen, Barcelona, Spain). Blood samples were also collected into tubes containing EDTA (Kangshi Zhejiang, China) and assayed for platelet count using an automatic animal blood analyzer (BC-2600vet, Mindray, Shenzhen, China). Lactate levels were determined by collecting 0.5 mL of rat blood with a heparinized syringe and analyzing it with a blood gas analyzer (GEM 3500, Wolfen, Barcelona, Spain). The supernatant Plasma was stored at −80°C refrigerator (Zhongke Meiling, Hefei, China) for subsequent testing. It is noteworthy that the duration of storage for all plasma samples was strictly limited to a maximum of 3 months to ensure the integrity and reliability of the samples for further testing.

### 2.7 Histopathological examination

After blood sampling, animals were sacrificed with an intraperitoneal injection of pentobarbital (100–150 mg/kg). The rats with no breathing and heartbeat for more than 5 min were identified with death. After euthanasia, the heart, lung, liver, kidney and intestine were collected, washed three times with normal saline, fixed in 10% formalin for 24 h, dehydrated using alcohol, embedded in paraffin, sectioned to a thickness around 5 μm, stained with hematoxylin and eosin, and sealed with an automatic sealing machine (CV5030, Leica, Wetzlar, Germany). Pathologists summed up the number of microthromboses in five randomly selected visual fields on each slice, which they examined under a light microscope (BMC100, Phoenix, Jiangxi, China). At a magnification of ×400, a total of five random fields of view were assessed for each organ to evaluate the presence of thrombi within the tissue sections.

### 2.8 Proteomics analysis

#### 2.8.1 Protein extraction and quantification

Samples were retrieved from the −80°C ultra-low temperature freezer (Haier, Qingdao, China) and subjected to centrifugation at 12,000 g for 10 min at 4°C. The resulting supernatant was meticulously transferred to new centrifuge tubes. A 5 μL aliquot from each protein sample was utilized to determine protein concentration using the BCA Assay kit (Beyotime, Zhejiang, China). Subsequently, the protein content of the samples was evaluated using SDS-PAGE to ensure precise quantification (Matsumoto et al., 2019).

#### 2.8.2 Protein digestion

An equal amount of protein from each of the 12 samples was selected for trypsin digestion. Initially, lysis buffer was added to normalize the protein volume across all samples. Proteins were then reduced by the addition of dithiothreitol to a final concentration of 5 mmol/L and incubated at 56°C for 30 min. Alkylation was performed by adding iodoacetamide to a final concentration of 11 mmol/L, followed by a 15-min incubation at room temperature in the dark. The alkylated proteins were transferred to ultrafiltration tubes and centrifuged at 12,000 g for 20 min at room temperature. The supernatant was collected, and the urea was exchanged three times with 8 M urea, followed by three exchanges with the replacement buffer. Trypsin was added at an enzyme-to-protein ratio of 1:50, and the samples were digested overnight. On the following day, peptides were collected by centrifugation at 12,000 g for 10 min at room temperature.

#### 2.8.3 Liquid chromatography-mass spectrometry analysis

Peptides were dissolved in HPLC mobile phase A, and chromatographic separation was conducted using the Vanquish Neo ultra-high performance liquid chromatography system (Thermo Scientific, United States). Mobile phase A consisted of an aqueous solution containing 0.1% formic acid and 2% acetonitrile, while mobile phase B was an aqueous solution containing 0.1% formic acid and 90% acetonitrile. The HPLC gradient was programmed as follows: 0–16 min, 7%–20% B; 16–24 min, 20%–32% B; 24–27 min, 32%–80% B; 27–30 min, 80% B, with a flow rate of 500 nL/min. Samples were ionized in the Nanospray Ion Source (NSI) and analyzed using an Orbitrap Exploris 480 mass spectrometer (Thermo Scientific, Massachusetts, United States), with data acquisition performed in DIA mode. The DIA data were searched using the Pulsar search engine embedded in Spectronaut (v17) against the Rattus_norvegicus_10116_PR_20230103.fasta database (47,945 sequences), with a reverse database to calculate the false discovery rate. The false discovery rate for protein, peptide, and protein identification was set at 1%. Post-database search, further data filtering was conducted to ensure high-quality results, with criteria including a protein FDR of 1% and at least one unique peptide for protein identification.

#### 2.8.4 Bioinformatics analysis

A comprehensive suite of quality control evaluations was applied to the mass spectrometry results to ensure data integrity. This was followed by protein quantification, reproducibility analysis, intensity value distribution analysis, and DEPs screening. The criteria for DEPs screening were set as follows: a significant upregulation threshold at a fold change (FC) > 1.5 and P < 0.05, and a significant downregulation threshold at FC < 1/1.5 and P < 0.05. The selected DEPs were subjected to KEGG functional classification, followed by GO enrichment analysis and KEGG pathway enrichment analysis. Ultimately, the DEP database IDs or protein sequences were compared with the STRING (v.11.0) protein-protein interaction database, and the differential protein interaction network relationships were elucidated based on a confidence score >0.7 (Nepusz et al., 2012).

#### 2.8.5 Parallel reaction monitoring (PRM) targeted mass spectrometry analysis

PRM targeted mass spectrometry analysis was performed on three groups of plasma samples to verify the selected differential proteins. The main steps include: (1) Extraction of proteins and subsequent enzymatic digestion, as delineated in reference (Wiśniewski et al., 2009); (2) Liquid chromatography-tandem mass spectrometry (LC-MS/MS) analysis (Aptbio, Shanghai, China): The peptide fractions were solubilized in solvent A of the liquid chromatography mobile phase. Chromatographic separation was executed on an EASY-nLC 1,200 ultra-high-performance liquid chromatography (UHPLC) platform (Thermo Scientific, Massachusetts, United States). Solvent A comprised an aqueous solution with 0.1% formic acid and 2% acetonitrile, whereas solvent B consisted of an aqueous solution with 0.1% formic acid and 90% acetonitrile. The gradient elution profile for the liquid chromatography was meticulously calibrated as follows: from 0 to 40 min, a linear gradient from 6% to 20% B; from 40 to 52 min, from 20% to 28% B; from 52 to 56 min, from 28% to 80% B; and from 56 to 60 min, isocratic at 80% B, with a flow rate of 500 nL/min. The peptides, once ionized, were directed into the NSI and subjected to analysis via a Q Exactive HF-X mass spectrometer (Thermo Fisher Scientific, Massachusetts, United States), employing Data-Independent Acquisition (DIA) mode for data collection. (4) Database searching and data processing: The DIA data garnered from this experiment were interrogated using MaxQuant software version 1.6.15.0, against the *Rattus norvegicus* protein database (Rattus_norvegicus_10116_PR_20230103.fasta), encompassing 47,945 sequences. Subsequent data refinement was performed employing Skyline version 21.2 (Schilling et al., 2015).

### 2.9 Enzyme-linked immunosorbent assay (ELISA)

Plasma samples were subjected to centrifugation at 3000 rpm for a duration of 10 min to achieve a clear separation. Subsequently, the samples or standards were aliquoted into the designated wells of the ELISA plate. The ELISA kits were utilized for the quantification of rat plasma levels of TM, TSP-1, vWF, and PAI-1. Adhering to the manufacturer’s guidelines, a sequence of procedural steps was meticulously executed, encompassing thorough mixing, incubation, washing, antibody addition, and colorimetric development. The optical density at 450 nm was subsequently measured using the MB-530 plate reader (Huisong, Shenzhen, China).

### 2.10 Statistical analysis

Statistical analyses were conducted using SPSS version 26.0 (IBM Corp., Armonk, NY, United States) and GraphPad Prism version 8.4 (GraphPad Software, La Jolla, CA, United States). The normality of the quantitative data was assessed using the Shapiro-Wilk test. One-way analysis of variance (ANOVA) was employed to evaluate differences among the three groups, followed by the least significant difference (LSD) test for *post hoc* pairwise comparisons. A p-value of less than 0.05 was considered to indicate statistical significance.

## 3 Results

### 3.1 The impact of pre-administration of SMY on exercise performance and coagulation function in EHS rats

**FIGURE 1:**
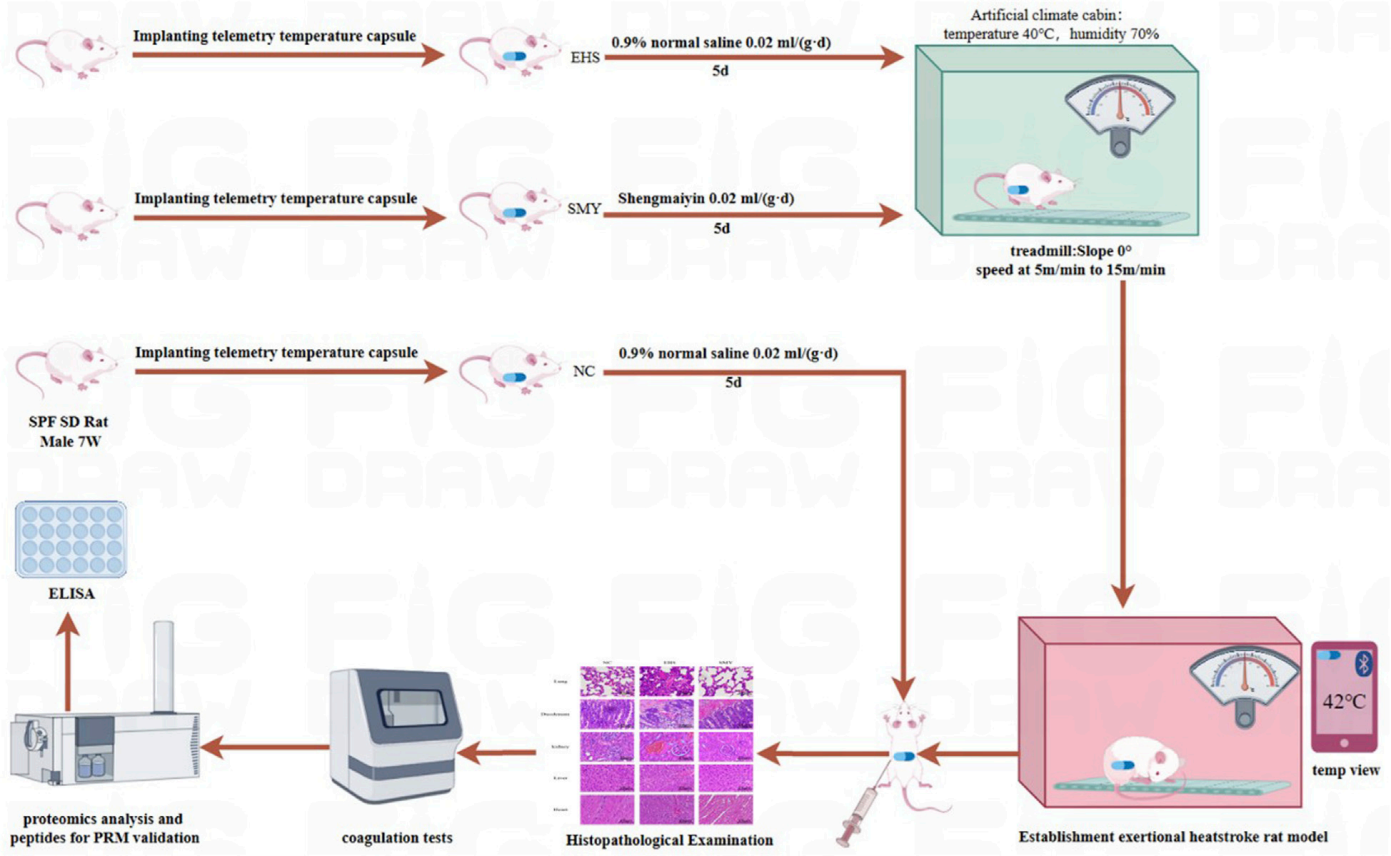
Establishment of EHS Model in rats.

**FIGURE 2:**
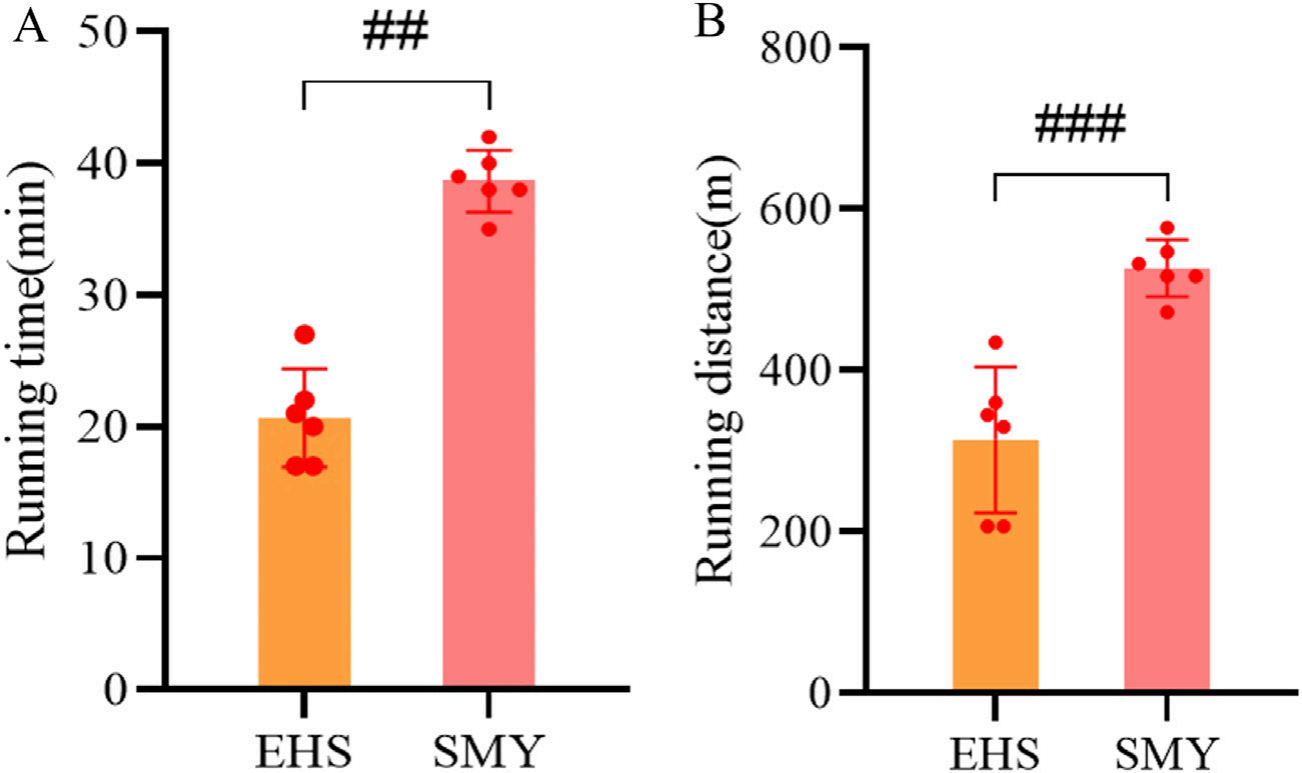
Motor performance parameters of rats in the SMY group and EHS group. **(A)**: running time; **(B)**: running distance.

The comprehensive experimental framework for the rodent study is depicted in Figure 1. Notably, the rats administered with SMY exhibited a markedly extended running distance and duration upon reaching a core body temperature of 42°C, surpassing those of the EHS group with statistical significance (*P* < 0.05), as illustrated in Figures 2A, B.

**FIGURE 3:**
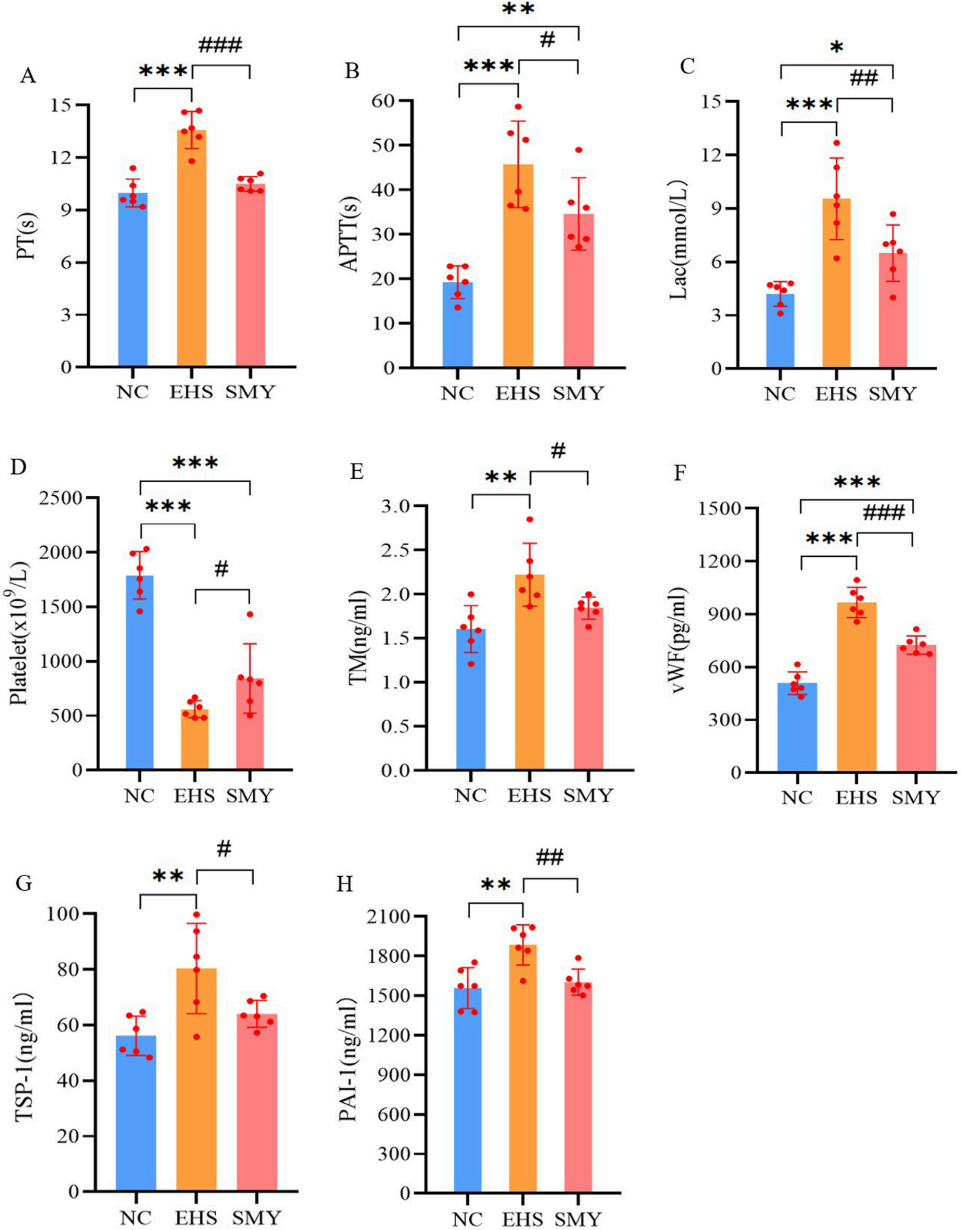
The impact of pre-administration of SMY on the coagulation function in EHS rats. **(A)**: prothrombin time (PT); **(B)**: activated partial thromboplastin time (APTT); **(C)**: blood lactic acid (Lac); **(D)**: platelet count; **(E)**: serum thrombomodulin (TM); **(F)**: von Willebrand factor (vWF); **(G)**: thrombin sensitive protein-1 (TSP-1); **(H)**: plasminogen activator inhibitor-1 (PAI-1).^*^ *p* < 0.05; ^**^ *p* < 0.01; ^***^ *p* < 0.001 vs. the NC group; ^#^ *p* < 0.05; ^##^ *p* < 0.01; ^###^ *p* < 0.001 vs. the EHS group.

In contrast to the NC group, the EHS group demonstrated a pronounced prolongation in PT and APTT, a significant rise in serum lactate levels, and a substantial decrease in platelet count, all of which were statistically significant (*P* < 0.05). Conversely, the SMY group, when compared to the EHS group, showed a reduction in PT and APTT, a decrease in lactate levels, and an increase in platelet count, with all these differences being statistically significant (*P* < 0.05), as detailed in Figures 3A–D.

Furthermore, the EHS group exhibited significantly higher plasma levels of TM, vWF, TSP-1, and PAI-1compared to the NC group, with all differences reaching statistical significance (*P* < 0.05). In comparison with the EHS group, the SMY group presented with significantly reduced plasma levels of TM, vWF, PAI-1, and TSP-1, with all these differences being statistically significant (*P* < 0.05), as represented in Figures 3E–H.

### 3.2 Histopathological examination

**FIGURE 4:**
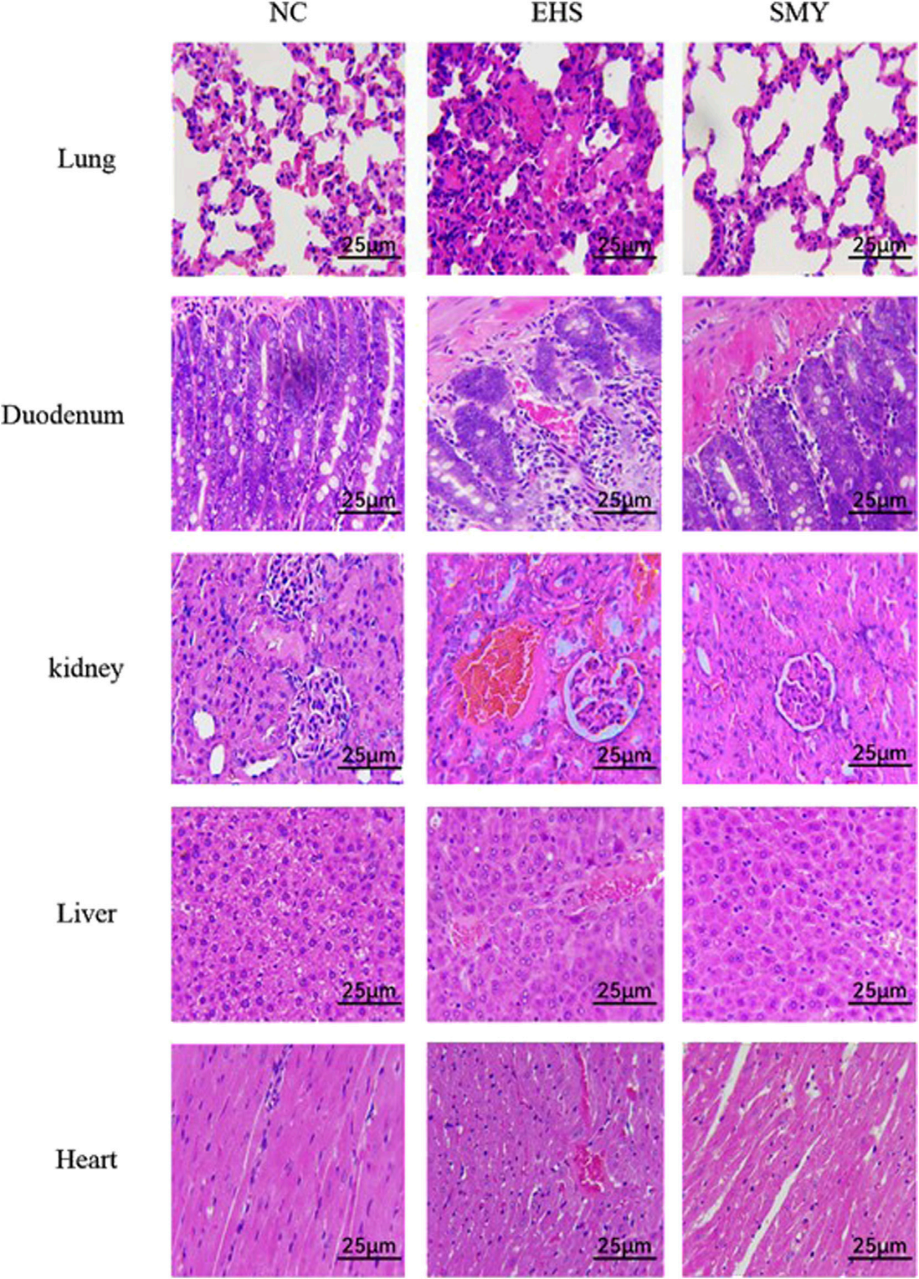
Histopathological Changes in Lung, Duodenum, Kidney, Liver, and Heart Tissues as Revealed by H&E Staining. Original Magnification ×400 Scale Bar: 25 μm.

The histopathological images of the lungs, duodenum, kidneys, livers, and hearts from rats in the NC, EHS, and SMY groups have been presented in Figure 4. The histological examination of tissues from rats in the NC group showed no signs of abnormalities. In contrast, rats from the EHS group exhibited moderate thickening of the alveolar walls in the lung tissue, accompanied by a reduction in alveolar cavity size, extensive neutrophil infiltration, and the presence of localized thrombi. The duodenum displayed dilation, with disruption of the duodenal villi, eosinophilic material accumulation at the tips, and cavitation. There was also detachment of the mucosal epithelium from the lamina propria and the formation of micro-thrombi. Renal histopathology showed congestion of the glomerular capillaries and renal interstitium, with micro-thrombi formation, significant dilation of the renal tubular lumens, and the presence of eosinophilic materials and neutrophil infiltration. In the liver, hepatocytes were markedly swollen, hepatic sinusoids were constricted, and pigment-laden Kupffer cells were observed. There was also extensive inflammatory cell infiltration and micro-thrombi formation. Myocardial cells in a small subset showed signs of necrosis, characterized by nuclear pyknosis and fragmentation, vacuolar degeneration, and micro-thrombi formation.

In the SMY group rats, the lung tissue exhibited mild, widespread thickening of the alveolar walls with scattered neutrophil infiltration. Duodenal tissue showed capillary congestion, minor mucosal epithelial cell necrosis, and desquamation. Renal tubular epithelial cells displayed mild edema, capillary congestion, and scattered inflammatory cell infiltration. Hepatocytes exhibited vacuolar degeneration, with some cells taking on a fatty appearance, and there was a minor degree of inflammatory cell infiltration. A small number of myocardial cells were observed to be swollen. Notably, no significant thrombi were detected in the histological sections of the various organs examined.

### 3.3 KEGG pathway annotation for DEPs in EHS rats pretreated with SMY

**FIGURE 5:**
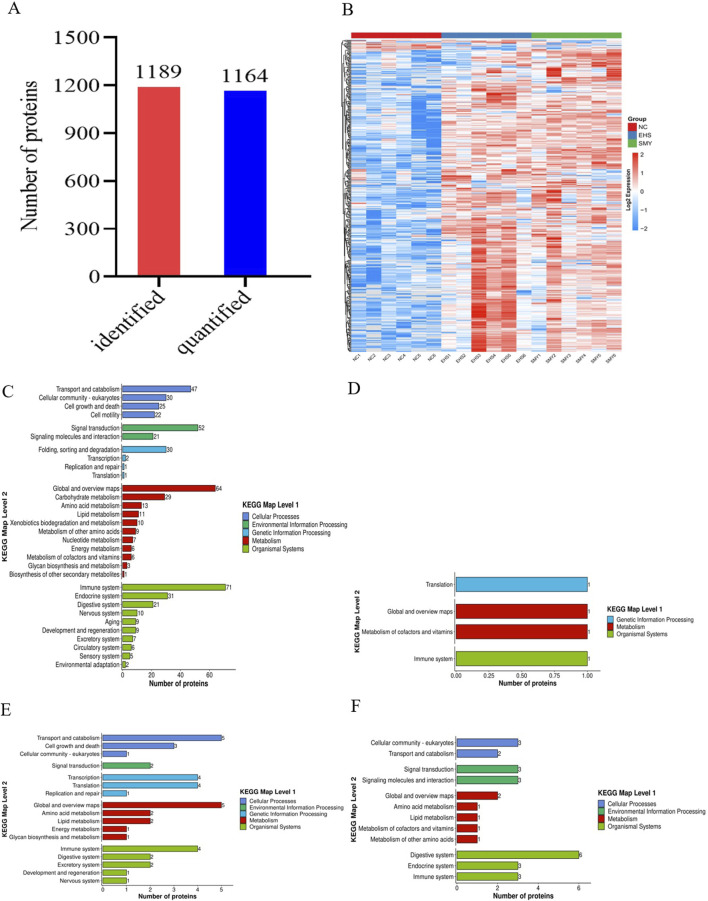
Comparative Proteomic Analysis of DEPs in EHS Rats Following Pretreatment with SMY. **(A)**: Overview of the Total Proteins Identified and Quantified; **(B)**: Heatmap Representation of DEPs; **(C)**: KEGG Pathway Analysis of Upregulated DEPs in EHS Rats Compared to Normal Controls (EHS vs. NC); **(D)**: KEGG Pathway Analysis of Downregulated DEPs in EHS Rats Compared to Normal Controls (EHS vs. NC); **(E)**: KEGG Pathway Analysis of Upregulated DEPs in SMY-Pretreated EHS Rats (SMY vs. EHS); **(F)**: KEGG Pathway Analysis of Downregulated Proteins in SMY-Pretreated EHS Rats (SMY vs. EHS).

In this study, a total of 1,164 proteins were quantified out of the 1,189 proteins that were identified (Figure 5A). The collective DEPs from all comparison groups were utilized to generate an expression heatmap (Figure 5B). These DEPs were further annotated with KEGG pathways. In comparison to the NC group, the DEPs in the EHS group were predominantly associated with cellular processes, environmental information processing, genetic information processing, metabolism, and organismal systems. The upregulated proteins were notably enriched in pathways involving transport and catabolism, cell community - eukaryotes, cell growth and death, cell motility, signal transduction, signaling molecules and interaction, folding, sorting, and degradation, as well as an overview of carbohydrate metabolism, immune system, endocrine system, and digestive system (Figure 5C). Conversely, the downregulated proteins were primarily found in pathways related to translation, an overview, cofactor and vitamin metabolism, and the immune system (Figure 5D).

In contrast to the EHS group, the DEPs in the SMY group were similarly categorized under cellular processes, environmental information processing, genetic information processing, metabolism, and organismal systems. The upregulated proteins in this group were predominantly concentrated in pathways associated with cell community - eukaryotes, transport and catabolism, signal transduction, signaling molecules and interaction, an overview, digestive system, endocrine system, and immune system (Figure 5E). On the other hand, the downregulated proteins were mainly associated with pathways involving transport and catabolism, cell growth and death, signal transduction, transcription, translation, an overview, amino acid metabolism, lipid metabolism, immune system, digestive system, and excretory system (Figure 5F).

### 3.4 Volcano plots and KEGG pathway enrichment analysis for DEPs

**FIGURE 6:**
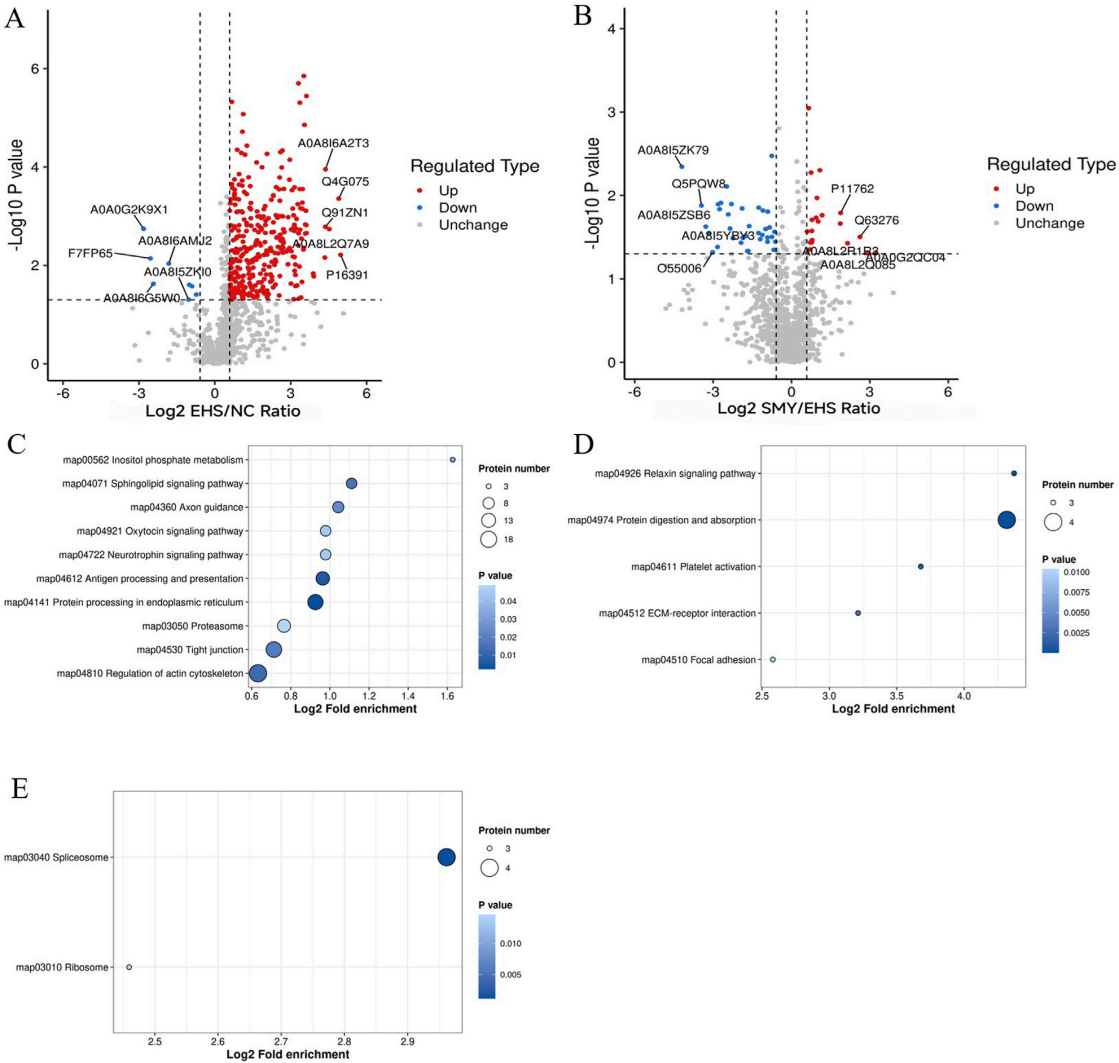
Volcano plots and KEGG pathway enrichment analysis for DEPs. **(A)**: Volcano plots of DEPs (EHS vs. NC); **(B)**: Volcano plots of DEPs (SMY vs. EHS); **(C)**: Bubble chart of KEGG pathway enrichment analysis of upregulated protein (EHS vs. NC); **(D)**: Bubble chart of KEGG pathway enrichment analysis of upregulated protein (SMY vs. EHS); **(E)**: Bubble chart of KEGG pathway enrichment analysis of downregulated protein (SMY vs. EHS).

In the EHS group, a comparative analysis with the NC group revealed 336 DEPs, with 328 proteins exhibiting upregulation and 8 showing downregulation. In contrast, when comparing the SMY group to the EHS group, 56 DEPs were identified, comprising 20 upregulated and 36 downregulated proteins. To graphically represent these DEPs, volcano plots were constructed for visual comparison across different groups (Figures 6A, B).

For the EHS group, when compared to the NC group, the upregulated signaling pathways predominantly encompassed inositol phosphate metabolism, sphingolipid signaling, axon guidance, oxytocin signaling, neurotrophin signaling, antigen processing and presentation, protein processing in the endoplasmic reticulum, proteasome, tight junction, and regulation of the actin cytoskeleton (Figure 6C). Notably, no signaling pathways were found to be downregulated in this comparison.

In contrast, when the SMY group was compared to the EHS group, the upregulated signaling pathways were primarily associated with relaxin signaling, protein digestion and absorption, platelet activation, ECM-receptor interaction, and focal adhesion (Figure 6D). Conversely, the downregulated pathways in this comparison were identified as the spliceosome and ribosome pathways (Figure 6E).

### 3.5 Analysis of protein interaction network for DEPs

**FIGURE 7:**
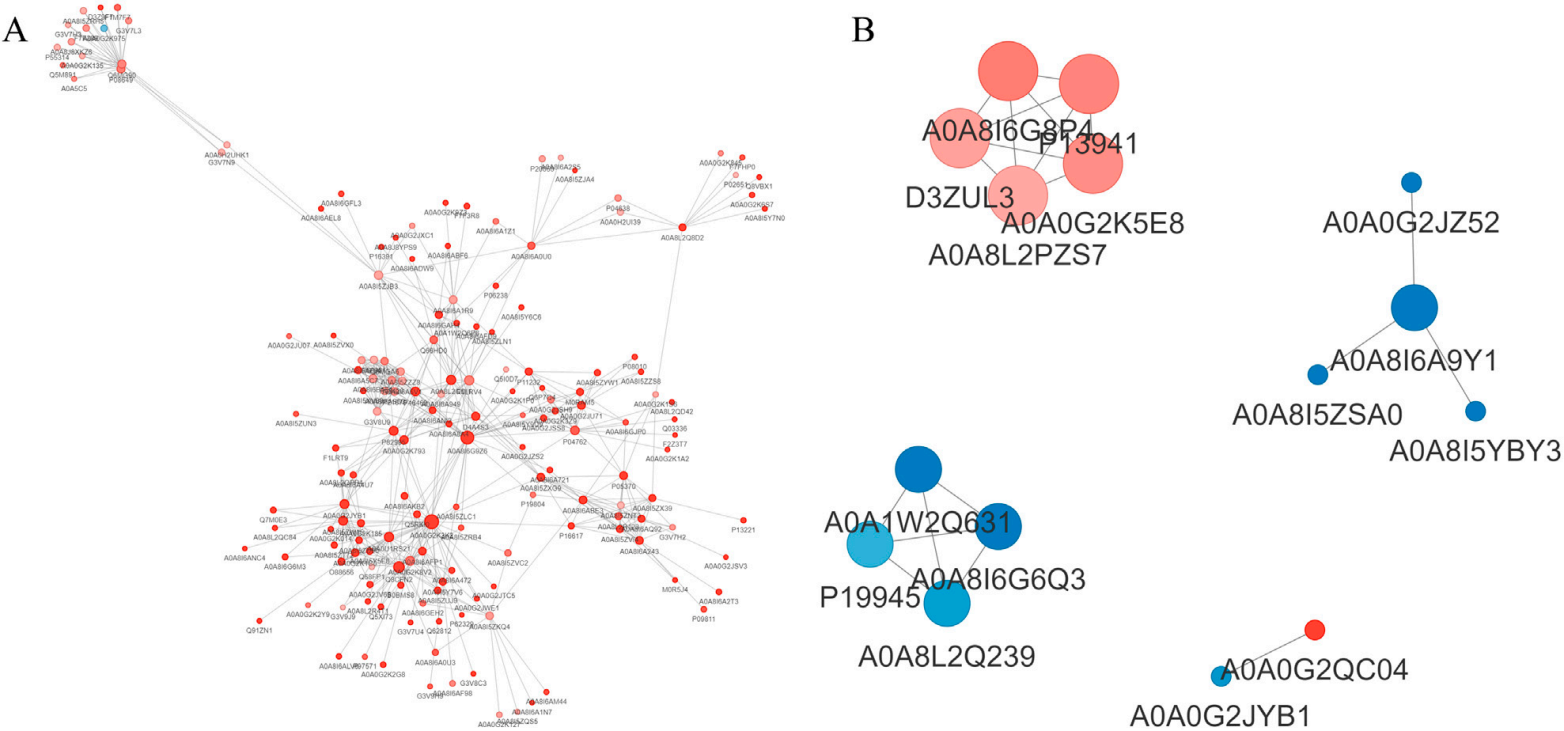
Analysis of protein interaction network. **(A)**: EHS vs. NC; **(B)**: SMY vs. EHS. Red circles represent upregulated proteins, while blue circles represent downregulated proteins, with deeper colors indicating a greater fold change, and the size of the circles reflecting the number of interacting proteins, with larger circles indicating more interacting proteins.

Upon comparison with the STRING protein interaction network database, interactions among DEPs with a confidence score exceeding 0.7 were identified. Subsequently, these DEP interaction networks were graphically represented using the R package “visNetwork” (Figures 7A, B).

In the SMY group, upregulated proteins such as AOA8I6G8P4 (Col1a1), P13941 (Col3a1), D3ZUL3 (Col6a1), A0A0G2K5E8 (Col1a2), and AOA8L2PZS7 (Pcolce) exhibited enhanced interactions with other proteins. These interactions were predominantly associated with pathways including platelet activation, relaxin signaling, protein digestion and absorption, extracellular matrix (ECM)-receptor interaction, and the PI3K-Akt signaling pathway.

Conversely, downregulated proteins in the SMY group, including A0A8I6A9Y1 (U2af2), A0A1W2Q63 (Rps16), A0A8I6G6Q3 (Serbp1), P19945 (Rplp0), and A0A8L2Q239 (Rpl23), were found to have increased interactions with proteins primarily implicated in the spliceosome and ribosome complexes.

### 3.6 Verification of target DEPs by PRM and ELISA

**FIGURE 8:**
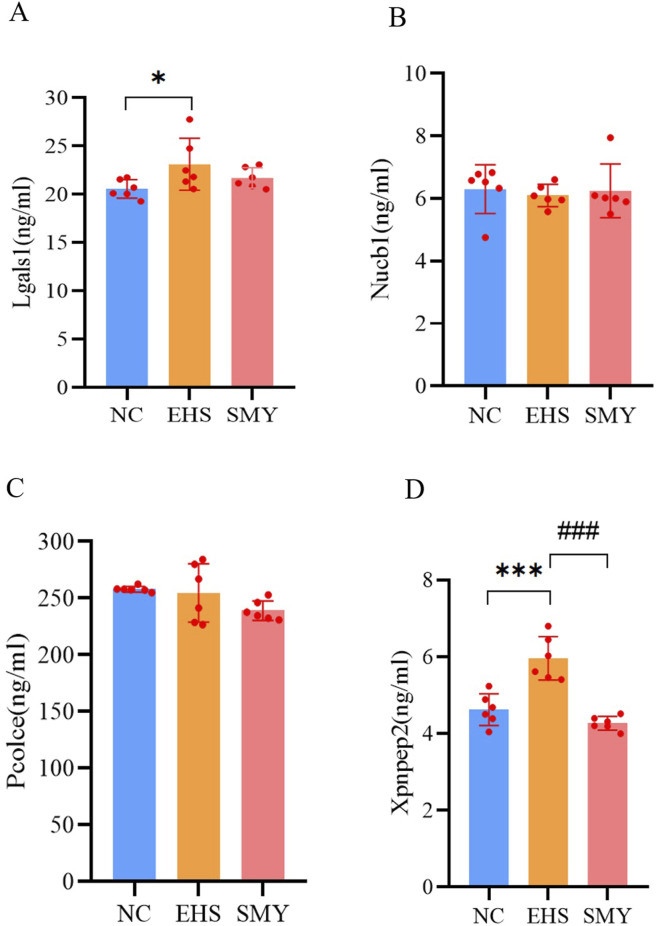
ELISA analysis for serum DEPs verified by PRM. **(A)**: lectin galactoside-binding soluble 1 (Lgals1); **(B)**: Nucleobindin-1 (Nucb1); **(C)**: Procollagen C-endopeptidase enhancer 1 (Pcolce); **(D)**: Xpnpep2. ^*^ *p* < 0.05; ^**^ *p* < 0.01; ^***^ *p* < 0.001 vs. the NC group; ^#^ *p* < 0.05; ^##^ *p* < 0.01; ^###^ *p* < 0.001 vs. the EHS group.

From the 226 DEPs between EHS and NC, 15 DEPs were further screened based on the criteria of SMY vs. EHS fold change (FC) > 1.5 or FC < 1/1.5, and *P* < 0.05. These 15 DEPs were verified using PRM, with quantification based on the peak areas of peptide fragment ions. The experimental design used 2 unique peptides per protein for quantification, but some proteins were only identified with 1 peptide due to sensitivity issues. Ultimately, 8 proteins were quantified (Table 1). The PRM verification criteria were: (1) if both PRM and proteomic FCs were >1.2, the expression was upregulated and the trends were consistent; (2) if both PRM and proteomic FCs were between 0.8–1.2, there was no difference and the trends were consistent; (3) if both PRM and proteomic FCs were <0.8, the expression was downregulated and the trends were consistent; (4) if one of the PRM or proteomic FCs was between 0.8-1.2, the absolute value of the log2 ratio of the two was calculated, and if it was <0.5, the trends were consistent, otherwise they were inconsistent. The PRM assay results demonstrated that the protein expression alterations of Nucb1, Pcolce, Lgals1, and Xpnpep2 were consistent with the proteomics findings. The ELISA experimental data revealed that the plasma concentrations of Lgals1 and Xpnpep2 in the EHS group rats were markedly elevated compared to the NC group (*P* < 0.05) (Figures 8A, D). Conversely, the plasma concentration of Xpnpep2 in the SMY group rats exhibited a significant reduction relative to the EHS group rats (P < 0.05) (Figure 8D). Notably, the plasma levels of Nucb1 and Pcolce remained consistent across all rats in the EHS, SMY, and NC groups, with no statistically significant variations observed (P > 0.05) (Figures 8B, C).

**TABLE 1:** Differentially expressed plasma proteins validated by PRM analysis.

UniProt ID	Protein description	Protein name	EHS/NC ratio PRM	EHS/NC ratio PRO	Consistency of DIA and PRM	SMY/EHS ratio PRM	SMY/EHS ratio PRO	Consistency of DIA and PRM
A0A0G2K1K3	Ganglioside GM2 activator	Gm2a	0.78851302	1.916948289	No	1.430400199	1.637097343	Yes
A0A0G2K9Z3	Nucleobindin-1	Nucb1	7.344622342	11.45025398	Yes	1.245339118	1.71780292	Yes
A0A8I5ZM13	Xaa-Pro aminopeptidase 2	Xpnpep2	1.7734453	3.073589113	Yes	0.753727701	0.435702534	Yes
A0A8I6A4U0	Ig-like domain-containing protein	ENSRNOG00000064589	1.620034386	2.03985662	Yes	0.535496111	0.592760309	Yes
A0A8I6AAG1	Ig-like domain-containing protein	AC109901.2	1.788049136	2.240486342	Yes	0.418612136	0.532380154	Yes
A0A8I6AC05	Ig-like domain-containing protein	ENSRNOG00000070455	1.561391774	2.855380951	Yes	0.891700954	0.503199957	No
A0A8L2PZS7	Procollagen C-endopeptidase enhancer 1	Pcolce	2.994805472	3.575152344	Yes	1.480570473	1.648518757	Yes
P11762	Galectin-1	Lgals1	1.535322795	3.115609812	Yes	2.551823912	3.678960116	Yes

## 4 Discussion

This study pioneers the discovery that the preemptive administration of SMY can forestall the coagulation dysfunction precipitated by exertional heatstroke. In the annals of traditional medicine, Astragalus, the cornerstone of SMY, is lauded for its capacity to invigorate vital energy and enrich blood and bodily fluids (Kan et al., 2022). Ophiopogon, the complementary component, is esteemed for its prowess in dispelling heat and stimulating fluid secretion, while Schisandra, the auxiliary element, is acknowledged for its role in nourishing yin and augmenting qi (Meng et al., 2023). Our study’s findings indicate that rats treated with SMY demonstrated a remarkable extension in both the duration and distance of their running endurance before reaching a critical core temperature of 42°C, underscoring SMY’s potential to bolster heat tolerance in rats, in alignment with the research by Lee et al. (Lee et al., 2005). Pathological evaluations unveiled that EHS rats exhibited thrombi within the microvasculature of the heart, lungs, liver, duodenum, and kidneys. Notably, rats that were administered SMY prior to heat exposure manifested pronounced cellular edema and inflammatory infiltration in these organs; however, microscopic examination did not reveal significant microthrombus formation.

Endothelial cell injury precipitated by heatstroke is recognized as a pivotal initiator of coagulopathy, as evidenced by our research which observed a pronounced elevation in biomarkers of endothelial injury, specifically plasma TM, vWF, and PAI-1 in rats subjected to EHS (Yu et al., 2024; Iba et al., 2024). This endothelial perturbation was paralleled by a notable extension in PT and APTT, alongside a marked escalation in plasma lactate concentrations. These observations suggest that endothelial injury during heatstroke triggers the coagulation cascade, leading to extensive microthrombi formation, microcirculatory disturbances, impaired organ perfusion, elevated lactate production, and ultimately, the onset of DIC (Maekawa et al., 2019). Furthermore, the considerable fluid loss incurred during heatstroke can diminish the effective circulating blood volume, potentially intensifying tissue hypoperfusion and lactate accumulation (Naß et al., 2021; Hashiguchi, 2017). Conversely, rats treated with SMY demonstrated a significant reduction in plasma levels of TM, vWF, TSP-1, and PAI-1, alongside a shortened APTT and an increase in platelet count, suggesting that SMY effectively attenuates the excessive consumption of coagulation factors and platelets in EHS rats. Literature suggests that TM augments the anticoagulant and antiplatelet activities of protein C (Korybalska et al., 2017); vWF triggers platelet activation and promotes platelet-mediated thrombosis (Giri et al., 2024); and PAI-1 curbs the activity of tissue plasminogen activator (t-PA), thus inhibiting fibrinolysis (Zhu et al., 2020). These observations collectively propose that SMY may ameliorate endothelial cell injury and coagulation disorders in EHS rats by modulating these critical pathways.

Proteomic analysis has elucidated the mechanisms underlying the ameliorative effects of SMY on coagulation disorders in rats with EHS. Among the 1,189 proteins identified, 56 DEPs were linked to SMY’s protective influence against HIC. The targeted proteomic profiling using PRM assays substantiated the initial proteomics data, particularly highlighting the altered expression of Nucb1, Pcolce, Lgals1, and Xpnpep2. Subsequent ELISA validation confirmed a pronounced increase in plasma Xpnpep2 levels in EHS rats, an effect that was effectively mitigated by pre-treatment with SMY.

XPNPEP2, a glycosylphosphatidylinositol (GPI)-anchored membrane-bound aminopeptidase, is composed of 673 amino acids and has a molecular mass of 75.5 kDa (Motta et al., 2023). This strategically positioned enzyme on the extracellular surface of the vascular endothelial cell membrane is ubiquitous across all human tissues (Hooper and Turner, 1988; Sprinkle et al., 1998). It selectively cleaves N-terminal Xaa-Pro bonds, thereby removing N-terminal amino acids, and is particularly renowned for its capacity to degrade bradykinin and a spectrum of neuropeptides. Bradykinin, the terminal product of the contact activation system, is derived from a cascade involving factor XII (FXII), plasma prekallikrein (PPK), and high molecular weight kininogen (HMWK). The activation of FXII catalyzes the conversion of prekallikrein into kallikrein, which then degrades HMWK to produce bradykinin (Colman and Schmaier, 1997). Bradykinin modulates neutrophil trafficking through the B1 receptor (BKB1R) and mediates vasodilation and increased microvascular permeability via the B2 receptor (BKB2R) (Weidmann et al., 2017; Stuardo et al., 2004). During periods of intense physical activity under high-temperature and high-humidity conditions, the human body secretes bradykinin to facilitate vasodilation of cutaneous blood vessels, thereby enhancing heat dissipation (Marceau et al., 2020; Lagneux et al., 1998). Simultaneously, there is a significant release of endogenous XPNPEP2, which degrades bradykinin. A reduction in bradykinin levels may impair the efficiency of vasodilation for heat dissipation, potentially precipitating heatstroke. Additionally, it impacts the reabsorption of sodium ions by the kidneys, exacerbating internal environment disorders and kidney damage (Matafora et al., 2021). The inhibition of XPNPEP2 has been demonstrated to elevate endogenous bradykinin levels, thereby improving cardiovascular dilation function and mitigating cardiac injury associated with ischemia/reperfusion (Erşahin et al., 2005). Furthermore, an upregulation of XPNPEP2 expression in the lungs of COVID-19 patients, aligning with the “cytokine storm” hypothesis, has been documented (Shansky et al., 2024). Consequently, the downregulation of Xpnpep2 by SMY may constitute a pivotal mechanism through which SMY confers protective effects on coagulation function in EHS rats.

The present study acknowledges several limitations that merit attention: Firstly, while we sourced the oral liquid of SMY from a uniform batch to ensure interventional uniformity, we did not perform a chemical analysis of the medication’s constituents. Secondly, this investigation has, for the first time, demonstrated that SMY can ameliorate thrombus formation and vascular endothelial cell injury associated with heatstroke. Our proteomics findings suggest that the suppression of Xpnpep2 expression is a key mechanism by which SMY exerts its effects, although a detailed exploration of the underlying mechanisms remains to be conducted. Thirdly, there is a need for further research to elucidate the expression patterns and functional roles of immunoglobulin-like domain-containing proteins, including ENSRNOG00000064589 and AC109901.2. Lastly, the study’s scope was confined to animal models, and the clinical applicability of our findings necessitates confirmation through clinical trials.

## 5 Conclusion

In summary, the pre-administration of SMY can alleviate vascular endothelial injury and mitigate coagulation dysfunction by modulating the plasma protein expression in heatstroke rats, with the downregulation of Xpnpep2 expression potentially being one of its primary mechanisms.

## Data Availability

The datasets used and/or analyzed during the current study are available from the corresponding author on reasonable request.

## Abbreviations

APTT, activated partial thromboplastin time; DEPs, differentially expressed proteins; EHS**,** exertional heatstroke; ELISA, Enzyme-Linked Immunosorbent Assay; HIC, heatstroke-induced coagulopathy; HMWK, high molecular weight kininogen; Lac, lactic acid; Lgals1, lectin galactoside-binding soluble 1; NC, normal control; Nucb1, Nucleobindin-1; PAI-1, plasminogen activator inhibitor-1; Pcolce, Procollagen C-endopeptidase enhancer; PPK, plasma prekallikrein; PRM, parallel reaction monitoring; PT, prothrombin time; SMY, Shengmaiyin; TM, thrombomodulin; TSP-1, thrombospondin-1; vWF, von Willebrand factor.
